# 

**Published:** 1889-05-11

**Authors:** 


SATURDAY, MAY 11th, 1889.

Lord Mayor Whitehead and the Working Classes 79

The Hospital Annual - 80

The Countess of Dufferin's Fund.—The National Association for Supplying Female Medical Aid to the Women of India - 81

The Student's Column.—Edinburgh University Medical Examination - 82

The Doctor's Pulpit.—Man and his Enemies - 83

The "Universal Review" for April - 84

A Woman's Clothes-Philosophy. By Xantippe Teufelsdrockh - 85

Mr. Gladstone and the Prince of Wales - 85

Notes and News - 86

Everybody's Page - 88

Annotations.—Medical Authority—Vaccination on its Trial—Moral Effects of Anæsthetics - 89

Student Life in Edinburgh. By A. G. B. Atkinson, B.A. 90

"Her Heart's Mistake." By E. D. Cumming, Author of "An Ordeal by Silence" - 91

A Great American Hospital - 92

For Women by Women - 93

Amusements.—Scraps and Gleanings - 94

EXTRA SUPPLEMENT—THE NURSING MIRROR.

En Passant.—A Bazaar by Nurses—Nursing in America—Clapham Maternity Charity—Halifax Infirmary—Rest for Newcastle Nurses—Recreation for Nurses—Army Nursing xix
Nursing in the Soudan.—On Duty Again - xx
Hail the First Thousand - xx
Our Friends Abroad - xxi
Everybody's Opinion.—Nursing at Nice—Torbay Hospital—Uniforms and Infection—Discontent—Almshouses—Private Nurses - xxi
Appointments—Vacancies—Notes and Queries - xxii

